# Water-Dispersible CsPbBr_3_ Perovskite Nanocrystals with Ultra-Stability and its Application in Electrochemical CO_2_ Reduction

**DOI:** 10.1007/s40820-021-00690-8

**Published:** 2021-08-12

**Authors:** Keqiang Chen, Kun Qi, Tong Zhou, Tingqiang Yang, Yupeng Zhang, Zhinan Guo, Chang-Keun Lim, Jiayong Zhang, Igor Žutic, Han Zhang, Paras N. Prasad

**Affiliations:** 1 Key Laboratory of Optoelectronic Devices and Systems of Ministry of Education and Guangdong Province, Institute of Microscale Optoelectronics, Shenzhen University, Shenzhen, 518060 People’s Republic of China; 2 Institute for Lasers, Photonics, and Biophotonics, Department of Chemistry, University at Buffalo, State University of New York , Buffalo, NY 14260 USA; 3 Department of Physics, University at Buffalo, State University of New York, Buffalo, NY 14260 USA; 4 Department of Chemical and Materials Engineering, School of Engineering, Nazarbayev University, Nur-Sultan City, 010000 Kazakhstan; 5 Jiangsu Key Laboratory of Micro and Nano Heat Fluid Flow Technology and Energy Application, School of Physical Science and Technology, Suzhou University of Science and Technology, Suzhou, Jiangsu 215009 People’s Republic of China

**Keywords:** CsPbBr_3_ nanocrystals, Water-dispersible, Ultra-stability, Electrochemical CO_2_ reduction

## Abstract

Thanks to the excellent optoelectronic properties, lead halide perovskites (LHPs) have been widely employed in high-performance optoelectronic devices such as solar cells and light-emitting diodes. However, overcoming their poor stability against water has been one of the biggest challenges for most applications. Herein, we report a novel hot-injection method in a Pb-poor environment combined with a well-designed purification process to synthesize water-dispersible CsPbBr_3_ nanocrystals (NCs). The as-prepared NCs sustain their superior photoluminescence (91% quantum yield in water) for more than 200 days in an aqueous environment, which is attributed to a passivation effect induced by excess CsBr salts. Thanks to the ultra-stability of these LHP NCs, for the first time, we report a new application of LHP NCs, in which they are applied to electrocatalysis of CO_2_ reduction reaction. Noticeably, they show significant electrocatalytic activity (faradaic yield: 32% for CH_4_, 40% for CO) and operation stability (> 350 h).

**Supplementary Information:**

The online version contains supplementary material available at 10.1007/s40820-021-00690-8.

## Highlights

- Water-dispersible CsPbBr_3_ nanocrystals (NCs) exhibit ultra-stability (>200 days) in water with only ~20% decline of the initial photoluminescence intensity.
- The as-prepared ultra-stable water-dispersible CsPbBr_3_ NCs showed high electrocatalysis activity (faradaic yield: 32% for CH_4_, 40% for CO) and operation stability (>350 h) for CO_2_ reduction reaction.

**Supplementary Information:**

The online version contains supplementary material available at 10.1007/s40820-021-00690-8.

## Graphical abstract

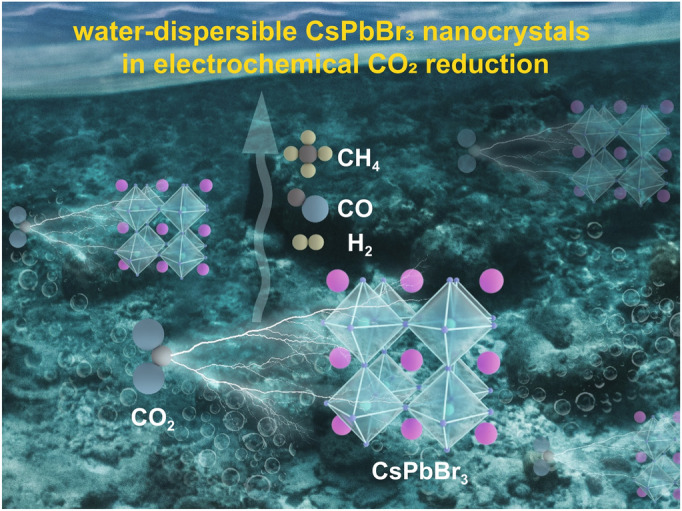

## Introduction

Electrochemical or photochemical reduction of carbon dioxide (CO_2_) could efficiently recycle the greenhouse gas back to fuels [1–3]. However, the existing perovskite catalysts are either inefficient or unstable. In addition, a weak binding interaction between the catalyst and the intermediates gives rise to high overpotential or slows the electron transfer kinetics, finally resulting in low-exchange current densities and turnover frequencies [4]. Both of these metrics depend on the intrinsic electronic properties of the catalyst, and also on the size effect, for example, bulk vs. nanostructures, thus current research hotspots are single-atom catalysts and also nanocrystals (NCs) catalysts [5, 6]. The emergence of lead halide perovskites (LHPs) substantially accelerates the development of material science [7–9]. Their outstanding optoelectronic properties, such as high photoluminescence (PL) quantum yield (PLQY), exceptional defect tolerance, long carrier diffusion lengths, lead to their wide applications in high-performance optoelectronic devices and photocatalysts [10–19]. However, the ionic nature of LHPs lead to their fast decomposition in the presence of water or moisture [20–22]. As a result, noticeable degeneration of the performance of LHP-based devices might arise when exposed to ambient condition [23]. Their poor stability against water largely restricts their applications in aqueous systems, such as electrocatalysis and biomedical labeling. Searching for a suitable strategy to synthesize water-stable or even water-dispersible LHPs is of significance to enhance the stability of LHP-based devices, as well as widen the field of LHP applications.

Till now, substantial efforts have been carried out to synthesize water-dispersible and -stable LHPs [24–31]. Especially, Wu et al. [28] obtained CsPbX_3_ NCs through a water-triggered phase transformation of Cs_4_PbX_6_ NCs. Interestingly, these CsPbX_3_ NCs exhibit improved stability against moisture due to the surface passivation effect. However, the as-prepared CsPbX_3_ NCs are still hexane-dispersible. Making core–shell structure is an effective approach to reduce the defects on the core surface and protect the core structure. Zhong et al. [32] reported a novel one-pot method for the synthesis of CsPbBr_3_@SiO_2_ core–shell nanostructure, which can greatly enhance the water-stability of CsPbBr_3_. Li et al. [33] synthesized CsPbBr_3_/polymer core–shell structure, which can reduce nonradiative current losses and improve quantum efficiency. However, these shell layers are almost insulating, which would result in poor charge transfer efficiency and reduce the performances of various devices. Li et al. [34] prepared CsPbBr_3_/TiO_2_ core–shell structure through the hydrolyzation of titanium butoxide (TBOT), more importantly, the TiO_2_ shell layer promotes enhanced water-stability and charge separation efficiency. However, the as-synthesized CsPbBr_3_/TiO_2_ core–shell structure possesses poor dispersibility in water. Recently, several publications reported that water-stable LHP NCs can be obtained through Lewis base vapor diffusion (LBVD) [35–37], aqueous synthesis protocol [30], or aqueous phase exfoliation methods [38]. However, their water-dispersibility or -stability is still not ideal for practical applications in an aqueous system. Moreover, the limited water stability of LHP leads to a largely overlooked application in electrocatalysis.

In this contribution, water-dispersible CsPbBr_3_ NCs were produced using a novel hot-injection method in a Pb-poor environment combined with a well-designed purification process. For the first time, CsPbBr_3_ NCs with good water dispersibility and excellent water stability (> 200 days) were realized. The water-dispersible CsPbBr_3_ NCs exhibit a high PLQY of 91%, which is much better than that of CsPbBr_3_ NCs dispersed in hexane (79%). Density-functional theory (DFT) calculation elucidates the ultra-stability of the water-dispersible CsPbBr_3_ NCs should be owned to the CsBr salt-rich environment, which reduces the surface defect density and prevents the structural degradation induced by water. Finally, the water-dispersible CsPbBr_3_ NCs were applied to the electrocatalysis of the CO_2_ reduction reaction (RR) in an aqueous system, resulting in high faradaic yields to CH_4_ (32%) and CO (40%).

## Experimental Section

### Chemicals

Lead bromide (PbBr_2_, 99.999% trace metals basis), cesium acetate (CsOAc, C_2_H_3_CsO_2_, 99.9% trace metals basis), zinc bromide (ZnBr_2_, 99.999% trace metals basis), 1-octadecene (ODE, C_18_H_36_, technical grade, 90%), oleylamine (OLA, C_18_H_37_N, technical grade, 70%), oleic acid (OA, C_18_H_34_O_2_, technical grade, 90%), and hexane (C_6_H_14_, 98%) were purchased from Sigma Aldrich. All chemicals were used without any further purification.

### Preparation of Cs-OA Solution

0.192 g (1 mmol) CsOAc and 2 mL OA were mixed in a flask, and the mixture was heated to 120 °C under atmosphere until all CsOAc was dissolved.

### Synthesis and Purification of Water-dispersible CsPbBr_3_ Nanocrystals

The water-dispersible CsPbBr_3_ nanocrystals were synthesized under a Cs- and Br-rich, which is a Pb-poor, environment. Briefly, 0.05 mmol PbBr_2_ and 0.95 mmol ZnBr_2_, which were used as Pb and Br sources (here, the ZnBr_2_ was used as the Br-source, which can provide a Br-rich environment, and Zn will not enter into the crystal lattice) [39, 40], were added into a 50 mL three-nicked flask that contained 20 mL ODE. The mixture was stirred and heated to 120 °C in an Ar atmosphere. 3 mL OA and OLA were used as surface ligands and added into the above solution. The temperature was raised to 180 °C after the complete dissolution of PbBr_2_ and ZnBr_2_, and then, 1 mL Cs-OA solution was swiftly injected. The reaction solution was cooled down to room temperature after 5 s reaction. The crude solution was directly centrifuged at 9000 rpm for 10 min. The suspension was discarded, and the precipitate was re-dispersed in 10 mL hexane. Subsequently, another centrifugation process was carried out at 9000 rpm for 10 min, the suspension and the precipitate (nonluminescent) were mixed separately with 2 mL of water under sonication, which resulted in CsPbBr_3_ nanocrystals with completely opposite dispersibility in hexane(marked as h-CsPbBr_3_) or water (marked as w-CsPbBr_3_), respectively. More detailed information can be found in Fig. S1.

### Electrode Preparation Process

The electrode was prepared by the spin-coating process described as following: CsPbBr_3_ NCs (w-CsPbBr_3_, without centrifugation process) thin film electrode was fabricated via spin-coating process. Before spin-coating, substrates were cleaned via ultrasonication in pure water, and after drying them, their surface was treated using a UV ozone cleaner (UV253E, Filgen) to decompose surface contaminants. For the electrochemical measurement, the edge and backside of the glassy carbon electrode were masked with caption tape to avoid the hydrogen evolution reaction. For spin-coating, the suitable concentration of CsPbBr_3_ NCs-5% Nafion solution ink was drop casted on the surface of the electrode. Spin-coating was conducted at 3000 rpm for 10 s and repeated 20 times until the loading amount reached 0.3 mg cm^−2^.

### CO_2_ RR Testing

The electrochemical measurements were performed using a Biologic SP-300 potentiostat. Ambient pressure CO_2_ electrolysis was carried out in a custom-made gas-tight electrochemical cell made of poly-carbonate and fitted with Buna-N O-rings built in our laboratory. The configuration of the electrochemical cell is such that the working electrode sits parallel with respect to the counter electrode to ensure a uniform potential distribution across the surface. The geometric surface area for both of the electrodes is 1.4 cm^2^. A Nafion 117 proton exchange membrane is used to separate the anodic and the cathodic compartments. Each of the compartments in this cell contains a small volume of electrolyte (2 mL each) to concentrate liquid products and therefore increase the detection limits. 0.1 M KHCO_3_ solution was used as the electrolyte, which was prepared by 1 h CO_2_ bubbling into 0.05 M K_2_CO_3_ solution (purified with Chelex resin to eliminate metallic impurities). Before starting the CO_2_ electrolysis, the electrolyte in the cathodic compartments was purged with CO_2_ for at least 15 min. During electrolysis, CO_2_ was constantly bubbled through the electrolyte at a flow rate of 5 sccm to prevent depletion of CO_2_ in the electrolyte and to allow the continuous analysis of gaseous products via a gas chromatograph. The flow rate of CO_2_ was controlled with a mass flow controller (Bronkhorst), and the gas was first humidified with water by passing it through a bubbler to minimize evaporation of the electrolyte. A platinum foil was used as the counter electrode, and an Ag/AgCl electrode (leak-free series) from Innovative Instruments, Inc. was used as the reference. Voltages were converted to the RHE scale by using a calibrated reference electrode as in the equation below:

$$E\left( {{\text{RHE}}} \right) \, = {\text{ EAg}}/{\text{AgCl }} + \, 0.0591*{\text{pH }} + \, E^{0} {\text{Ag}}/{\text{AgCl}}$$

### Product Analysis

For the analysis of gaseous products, a gas chromatograph (GC, SRI instruments) equipped with a HayeSep D porous polymer column, thermal conductivity detector, and flame ionization detector, was used. Ultra-high purity Ar (99.999%) was used as the carrier gas. The concentrations of gaseous products were determined using calibration curves from standard gas mixtures. During electrolysis, CO_2_ was constantly bubbled through the electrolyte to prevent depletion of CO_2_ in the electrolyte, and to allow continuous analysis of gaseous products via the GC.

### Characterization

The crystal structures and microstructures of the as-synthesized samples were examined using XRD with Cu K*α* radiation (Bruker D8 Advance powder X-ray diffractometer) and TEM (JEM-3100, JEOL, Japan). The optical properties of the as-synthesized samples were determined using a UV − vis spectrophotometer (UV − 3101PC, Shimadzu) with excitation provided by a 365 nm laser (RF-5301PC, Shimadzu) using CsPbBr_3_ NCs solutions. h-CsPbBr_3_ and w-CsPbBr_3_ NCs were separately kept in two sealed quartz cuvettes for the water-stability testing. In order to minimize the errors, the same positions of each quartz cuvettes were used during the testing. The PLQY was examined with a Hamamatsu C11347-12 Quantaurus-QY fluorescence spectrometer using CsPbBr_3_ NCs solutions. The blank solutions (*i.e.,* hexane and water for h-CsPbBr_3_ and w-CsPbBr_3_ NCs, respectively) were measured and named as references, then the PLQY of h-CsPbBr_3_ and w-CsPbBr_3_ NCs were received by deducting the references. The time-resolved PL lifetime was collected with a fluorescence spectrometer (FLS 980) using a 507 nm laser for excitation (EPL-510, Edinburgh Instruments Ltd) using CsPbBr_3_ NCs solutions. *ζ* potential data was collected using a Zetasizer Nano ZSE analyzer (Malvern, United Kingdom).

### Density-Functional Modeling Methods

The geometry optimization and electronic structure calculations were performed by using the projector augmented wave method (PAW) [41] based on the density-functional theory (DFT), as implemented in the Vienna ab initio simulation package (VASP) [42]. The Perdew-Burke-Ernzerhof generalized-gradient approximation (PBE- GGA) [43] is used to describe the exchange and correlation functional. All calculations were carried out with a plane-wave cutoff energy of 450 eV. For the calculation on the interaction between CsPbBr_3_ NCs and water molecule, a periodic (1 × 1) surface supercell is built with a vacuum layer of 20 Å, and the Monkhorst–Pack k-point grids of 9 × 9 × 1 are adopted for the first Brillouin zone integral. For the calculation on processes of CO_2_ RR at CsPbBr_3_ NCs, a periodic (2 × 2) surface supercell is built with a 20 Å vacuum layer, and Monkhorst–Pack k-point grids of 3 × 3 × 1 are adopted. The final stable atomic structures are obtained through full relaxation with a total energy tolerance of 10^−6^ eV. The van der Waals interaction functional with the method of Grimme (DFT-D3) [44] was employed in the calculations.

## Results and Discussion

### Synthesis and Characterization of CsPbBr_3_ NCs

**Fig. 1:**
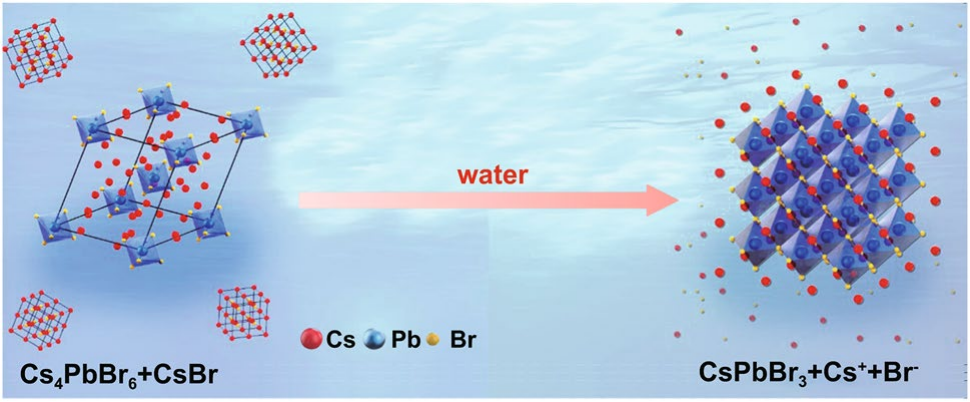
Schematic of the phase transformation from CsBr/Cs_4_PbBr_6_ to CsPbBr_3_

The synthesis process was carried out in a Pb-poor and Cs-/Br-rich environment through a hot-injection method (more details can be found in Experimental Section). In a Cs-/Br-rich environment, the expected products should be a mixture of CsBr/Cs_4_PbBr_6_. The crude solution exhibited white color without any visible PL. A well-designed purifying process was carried out to obtain water-dispersible CsPbX_3_ NCs from the crude solution, as shown in Fig. S1. The reaction solvents, including 1-octadecene (ODE), oleic acid (OA), and oleylamine (OLA), were separated from the crude solution by direct centrifugation. Then, a further centrifugation process was performed after re-dispersion of the precipitate in hexane. Both the suspension and the precipitate showed mixed phases of CsBr/Cs_4_PbBr_6_ (Fig. S2) without any PL. A phase transformation from Cs_4_PbBr_6_ to CsPbBr_3_ was observed after inducing of water (Fig. 1), the CsPbBr_3_ phase with strong PL emission emerged in both h-CsPbBr_3_ ( dispersed in hexane) and w-CsPbBr_3_ ( dispersed in water).

**Fig. 2:**
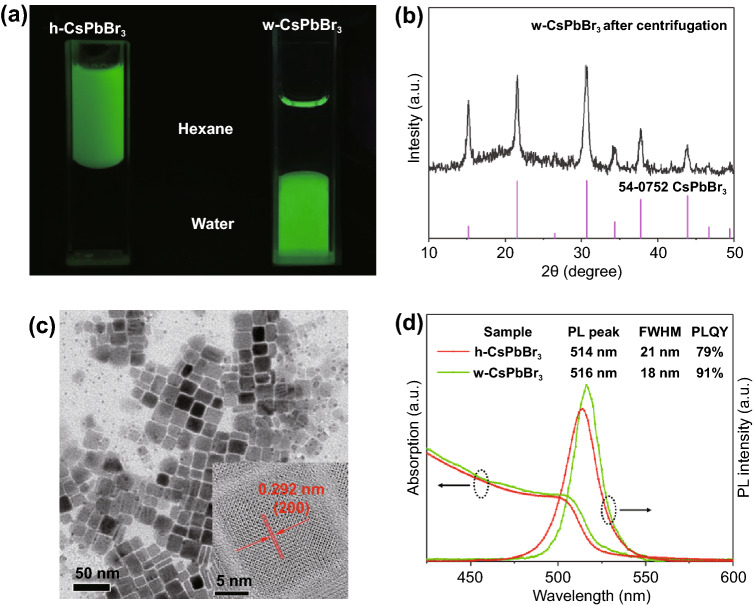
**a** Photographs of h-CsPbBr_3_ and w-CsPbBr_3_ NCs under UV light; XRD pattern (**b**) and TEM image (**c**) of w-CsPbBr_3_ NCs after centrifugation; **d** UV–vis absorption and PL spectra of w-CsPbBr_3_ NCs

Surprisingly, these NCs exhibited completely opposite dispersibility in hexane or water, as shown in Fig. 2a. In the preparation process of w-CsPbBr_3_, an organic layer emerged at the top of the water layer after sonication, which indicated the removal of the surface organic ligands. Fourier transform infrared (FTIR) spectroscopy further confirmed that most of the ligands had been removed. As shown in Fig. S3, the vibrational bands around 3000 cm^−1^ (C–H stretching) and below 2000 cm^−1^ (C = O/C = C stretching and C-H banding) belonging to oleylammonium, oleate, or octadecene [45] are almost disappeared. XRD patterns (Fig. S2) clearly showed the pure CsPbBr_3_ phase of h-CsPbBr_3_, while w-CsPbBr_3_ can be indexed as a mixture of CsBr and cubic CsPbBr_3_ phases [46, 47]. Furthermore, the pure cubic CsPbBr_3_ phase from w-CsPbBr_3_ had also been obtained after centrifuging at 13,000 rpm for 20 min (Fig. 2b), indicating the perfect water solubility of CsBr in w-CsPbBr_3_. Transmission electron microscopy (TEM) images of w-CsPbBr_3_ (after centrifugation) and h-CsPbBr_3_ NCs are shown in Figs. 2c and S4, both samples have uniform size distributions (19.49 and 8.77 nm for w-CsPbBr_3_ and h-CsPbBr_3_, respectively, Fig. S5), regular morphologies, and good crystallization. The interlattice distances of 0.292 and 0.419 nm matched well with the d-spacing of (200) and (110) crystal planes of cubic CsPbBr_3_. The optical properties of h-CsPbBr_3_ and w-CsPbBr_3_ NCs were further studied by UV–vis absorption and PL spectra, as shown in Fig. 2d. The absorption onset and the PL peak of h-CsPbBr_3_ NCs are determined at 519 and 514 nm, respectively, which are 2 nm lower than those of w-CsPbBr_3_ NCs (521 and 516 nm). Interestingly, w-CsPbBr_3_ NCs exhibited a narrower full width at half-maximum (FWHM) of 18 nm and a higher PLQY (91%) than those of h-CsPbBr_3_ NCs (21 nm and 79%, respectively).

**Fig. 3:**
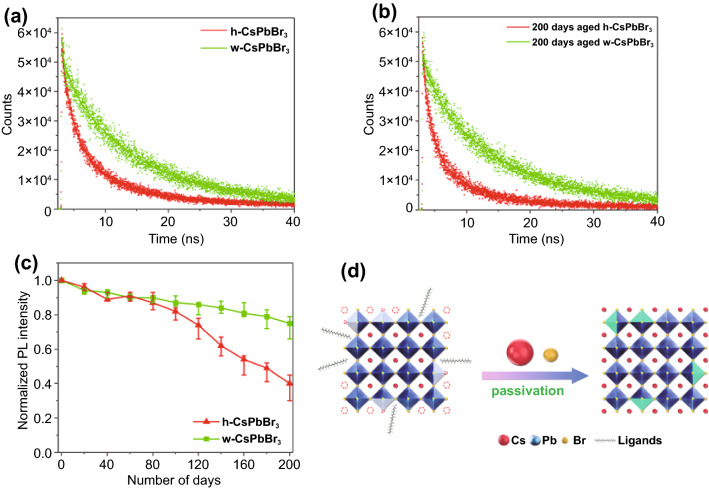
TRPL spectra of freshly prepared (**a**) and 200 days aged (**b**) h-CsPbBr_3_ and w-CsPbBr_3_ NCs, respectively; **c** PL stabilities of h-CsPbBr_3_ and w-CsPbBr_3_ NCs; **d** schematic of the surface passivation of CsPbBr_3_ NCs

The time-resolved PL (TRPL) shown in Fig. 3a indicated that w-CsPbBr_3_ NCs has a longer PL lifetime (10.27 ns) than that of h-CsPbBr_3_ NCs (4.49 ns), suggesting a lower surface defect density in w-CsPbBr_3_ NCs, which is attributable to the surface passivation effect induced by the Cs^+^ and Br^–^ ions in water [48]. In addition, the PL lifetime of 200 days aged w-CsPbBr_3_ NCs was 9.90 ns (Fig. 3b), which is close to the initial value (10.27 ns). In contrast, a significant decrease in the PL lifetime from 4.49 ns (initial) to 2.92 ns (200 days aged) was observed for h-CsPbBr_3_ NCs, which further revealed the lower surface defect density and improved stability of w-CsPbBr_3_ NCs. Figure 3c demonstrated the PL stability of h-CsPbBr_3_ and w-CsPbBr_3_ NCs. Unexpectedly, w-CsPbBr_3_ NCs received a better PL stability than that of h-CsPbBr_3_ NCs. w-CsPbBr_3_ NCs could maintain 80% of its initial PL intensity for more than 200 days. However, the PL intensity of h-CsPbBr_3_ NCs decreased rather rapidly after 100 days, and could only preserve about 40% of its initial PL intensity after 200 days. Surprisingly, when the freshly prepared w-CsPbBr_3_ NCs were centrifuged and re-dispersed in pure water, complete quenching of the PL emission was observed within 24 h. That is to say, the CsBr salt in water played a crucial role for the ultra-stability of w-CsPbBr_3_ NCs. It is well known that the CsPbX_3_ (X = Cl, Br, I) NCs are terminated by either CsX or PbX_2_, and passivated by oleylammonium ions (OLA^+^), which occupy the A-sites (h-CsPbBr_3_) [49]. The loss of OLA^+^ and the emergence of Br-vacancy would occur gradually over time (Fig. 3d), which leads to the degeneration of both the crystal structure and the optical properties. As the organic ligands on the surface of w-CsPbBr_3_ NCs had been removed after the washing process, the sufficient Cs^+^ and Br^−^ ions in the water can passivate the defects induced by the loss of OLA^+^ and Br^−^ ions (Fig. 3d). Therefore, we believe that the CsBr salt in water is essential for the better performance of w-CsPbBr_3_ NCs.

### Water Stability Mechanism

**Fig. 4:**
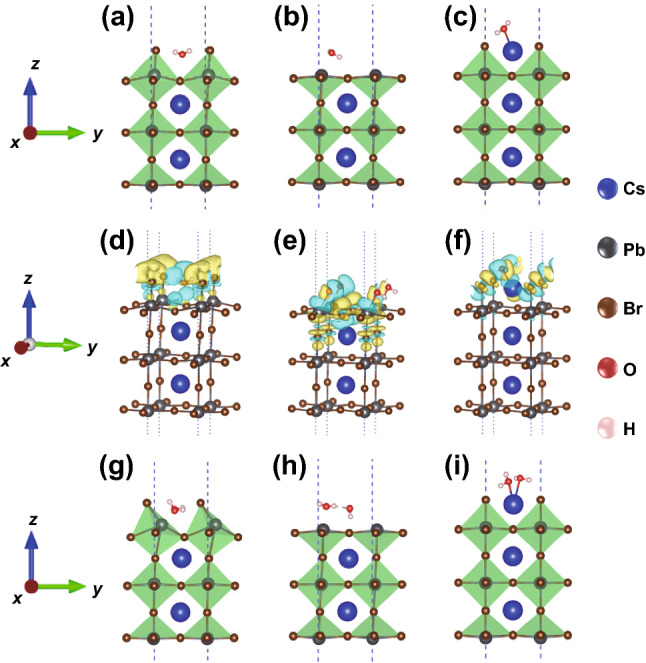
Relaxed polyhedral models of CsPbBr_3_ with (**a**) initial, **b** Br-vacancy, and (**c**) CsBr passivated structures after adsorption of one water molecule. **d**–**f** Diffrential charge density for (**a**–**c**), respectively. The yellow and cyan regions represent electron accumulations and depletions, respectively, where the isosurface value was set to 0.002 e Å^−3^. Relaxed polyhedral models of CsPbBr_3_ with (**g**) initial, (**h**) Br-vacancy, and (**i**) CsBr passivated structures after adsorbing two water molecules. Blue, gray, brown, red, and pink spheres represent Cs, Pb, Br, O, and H atoms, respectively

Generally, the stability of material can be evaluated on the basis of the robustness of the crystal and electronic structures against environment. Specifically, if the crystal and electronic structures of one material remain unchanged when interacting with the environment, it can be regarded as a stable material under this environment. In contrast, the material is unstable. Changes in the crystal structure can be directly obtained from the structure distortion after structural relaxations, and changes in electronic structure are usually reflected in the charge redistributions (known as charge transfer). Such crystal structure distortion and charge redistributions have been widely used to check the stability of perovskites in a specific environment using DFT calculations [50–52]. Here, we calculated the crystal structure distortions and charge redistributions of the CsPbBr_3_ after the adsorption of H_2_O molecules to elucidate the mechanism of CsBr-induced water-stability of w-CsPbBr_3_ NCs. It is well known that CsPbBr_3_ possesses a PbBr_2_ terminated surface (Fig. S6a, d) [43], the structure distortion of [PbBr_6_]^4−^ octahedra and octahedral cavities induced by the adsorption of a water molecule can be clearly observed, as shown in Figs. 4a and S7a. The deviations of the Pb − Br bond lengths and Pb − Br − Pb bond angles were listed in Table S1 and Fig. S7. Such structural distortions were also verified by calculating the charge distribution, which is described by the differential charge density of the system, Δ*ρ* = *ρ*(H_2_O/CsPbBr_3_) − *ρ*(H_2_O) − *ρ*(CsPbBr_3_), where *ρ*(H_2_O/CsPbBr_3_), *ρ*(H_2_O), and *ρ*(CsPbBr_3_) are the charge densities of the H_2_O/CsPbBr_3_, H_2_O, and CsPbBr_3_, respectively. As shown in Fig. 4d, Δ*ρ* is mainly distributed in the first layer of the [PbBr_6_]^4−^ octahedra, indicating a noticeable structural distortion of the [PbBr_6_]^4−^ octahedra on the surface. Figure S6b, e demonstrate the CsPbBr_3_ structure with a Br-vacancy, which is common due to the easy loss of Br^−^ ions. When one H_2_O molecule is absorbed, distinct distortions can be observed both at the surface (first layer) and the inner structure (second layer), as shown in Figs. 4b and S5b. In this case, Δ*ρ* not only existed in the first layer of [PbBr_6_]^4−^ octahedra, but also emerged in the second layer of [PbBr_6_]^4−^ octahedra, indicating that the water molecules can even induce the distortion to the [PbBr_6_]^4−^ octahedra inside (Fig. 4e). The appearance of CsBr can lead to a CsBr terminated surface, which produced CsBr passivation, as shown in Fig. S6c, f. With such a CsBr passivated surface, the [PbBr_6_]^4−^ octahedra were more stable even after the adsorption of water molecules, as shown in the relaxed structural geometries (Figs. 4c and S7c), where the structural deviations were minimal (Table S1). As seen in Fig. 4f, the charge transfer only existed around the Cs atoms on the surface, indicating that the water molecules had little effect on the [PbBr_6_]^4−^ octahedra. The CsBr terminated surface can be seen as a protective shell for the [PbBr_6_]^4−^ octahedra structures. Subsequently, two water molecules were further introduced onto the surface of various structures (Figs. 4g-i and S7d-f). Interestingly, the distortion of the PbBr_2_ terminated and Br-vacancy crystal lattice became more prominent compared to the results shown in Fig. 4a, b. No significant difference can be found for the CsBr passivated structure after adsorbing two water molecules, suggesting that the CsBr passivated structure was very stable. These results further revealed that the CsBr salt plays a crucial role for the ultra-stability of w-CsPbBr_3_ NCs in water.

In addition, we believe that the Cs^+^ and Br^−^ ions in water can form an electrical double layer (EDL) on the surface of w-CsPbBr_3_ NCs due to the large *ζ* potential (> 80 mV, Figs. S8 and S9) [53]. The stability of perovskite NCs in hexane highly relies on lipophilic ligands on the surface. In contrast, the high stability of w-CsPbBr_3_ NCs against water in this work is attributed to EDL, which is extensively believed to be the reason for the good dispersibility of colloids in water. Both Cs_4_PbBr_6_ and CsPbBr_3_ are composed of [PbBr_6_]^4−^ octahedrons and Cs^+^ ions. For Cs_4_PbBr_6_, [PbBr_6_]^4−^ octahedrons are completely separated from each other and surrounded by Cs^+^ ions, while the [PbBr_6_]^4−^ octahedrons in CsPbBr_3_ share all corners with Cs^+^ ions filling in voids [28]. Due to the high solubility of Cs^+^ ions in water, Cs_4_PbBr_6_ and CsPbBr_3_ NCs covered by lipophilic ligands show poor stability against moisture. The stability of Cs_4_PbBr_6_ is even worse than that of CsPbBr_3_ because of its incompact structure, and Cs_4_PbBr_6_ can transform to CsPbBr_3_ by stripping Cs^+^ and Br^−^ ions into water [28]. In this work, once we disperse the CsBr/Cs_4_PbBr_6_ NCs into water and sonication, surface ligands can be removed. In addition, the CsBr NCs will immediately be dissolved, and Cs^+^ ions will gradually leach from Cs_4_PbBr_6_ NCs and result in a negative charge of the remaining part of Cs_4_PbBr_6_, which in turn slows down the leaching of Cs^+^ ions. Additionally, the Br^−^ also begins to leach into the water to maintain electric neutrality, and the isolated [PbBr_6_]^4−^ octahedrons will share their corners. The [PbBr_6_]^4−^ framework with Cs^+^ ions filling into the voids is exactly the structure of CsPbBr_3_. At this moment, the newly-formed CsPbBr_3_ NCs are surrounded by high concentration Cs^+^ and Br^−^ ions. Cs^+^ ions have a high tendency to absorb on the surface of CsPbBr_3_ due to the intrinsic property of CsPbBr_3_. The absorption of Cs^+^ ions leads to positive charges at the surface, and then Br^−^ is dragged around the surface by electrostatic interaction. These two layers of Cs^+^ and Br^−^ ions form the EDL, and the first Cs^+^ ions are the potential determining ions. The EDL can move with the inner NCs in water, and there is a shear plane between the EDL and solution. *ζ* potential is just the potential at shear plane (Fig. S9). The large *ζ* potential of > 80 mV of w-CsPbBr_3_ NCs guarantees their perfect dispersion in water. More importantly, the large *ζ* potential can provide a high energy barrier so that the Cs^+^ and Br^−^ ions in the w-CsPbBr_3_ NCs will not penetrate through the EDL and leach into water (even though the concentration of CsBr is much lower than its solubility (1243 g L^−1^ at 25 °C)). Furthermore, the Cs^+^ ion layer on the surface of w-CsPbBr_3_ NCs can, in turn, improve the stability of [PbBr_6_]^4−^ framework, as demonstrated in Fig. 4. Therefore, w-CsPbBr_3_ NCs can maintain high stability in water. However, if the freshly prepared w-CsPbBr_3_ NCs were centrifuged and re-dispersed in pure water, the EDL on the surface of w-CsPbBr_3_ NCs will be destroyed. As a result, complete quenching of the PL emission could be observed within 24 h.

### Electrochemical Performance of CsPbBr_3_ NCs

**Fig. 5:**
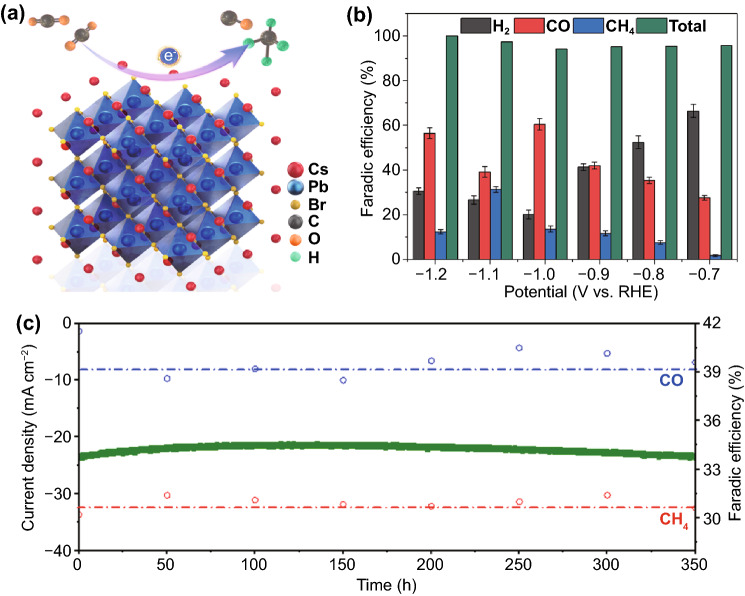
**a** Schematic of the w-CsPbBr_3_ NCs CO_2_ RR; **b** Faradic efficiencies for the w-CsPbBr_3_ NCs CO_2_ RR; **c** chronopotentiometry curve (long-term stability) of w-CsPbBr_3_ NCs in 0.1 M KHCO_3_ for 350 h

Colloidal NCs have emerged as promising materials for electrocatalytic applications due to the ability to tailor their properties through size modulation [54, 55]. However, because of the unstable and catalytically inert property of CsPbBr_3_, it has been commonly used as either a co-catalyst or a light capture agent during the catalytic reactions [19]. Here, for the first time, we have successfully prepared ultra-water-stable CsPbBr_3_ NCs and demonstrated their applications in electrocatalysis (Fig. 5a). The as-prepared w-CsPbBr_3_ NCs were measured as electrocatalysts for the CO_2_ RR in an H-cell using CO_2_-saturated 0.1 M KHCO_3_ as the electrolyte. The electrocatalytic reduction products were further confirmed by both gas chromatography (GC) and ^1^H nuclear magnetic resonance (NMR). Figure 5b showed the faradaic efficiencies (FEs) for the w-CsPbBr_3_ NCs from − 0.7 to − 1.2 V versus reversible hydrogen electrode (RHE). The main products of the catalyst were H_2_, CO, and CH_4_. At − 0.7 V, H_2_ was more than 70%, which indicated that the hydrogen evolution reaction should be dominant compared to the CO_2_ RR at this potential. From − 0.8 V, the CO_2_ RR mainly generated both CO and CH_4_; at − 1.1 V, the reaction yielded mostly CH_4_ (32%), and CO (40%). To the best of our knowledge, this result is the first report so far for the LHP NCs electrochemical CO_2_ RR. It also showed that our catalysts did not favour the production of methanol (CH_3_OH), which is one of the other CO_2_ hydrogenation products. Although many transition metals have the potential to produce both methane and methanol, the ionophilicity of the surface, as measured by the Oads binding energy, plays a critical role in determining the selectivity between these two products. Additionally, the catalyst also showed ultrahigh catalytic stability for CO_2_ RR with at least 350 h of long-term running for chronoamperometry (CA), as shown in Fig. 5c. Moreover, the crystal structure of the w-CsPbBr_3_ NCs after reaction, as well as its statistic current long-term stability had also been characterized, as shown in Fig. S10. The results further confirmed the ultrahigh catalytic stability of the w-CsPbBr_3_ NCs. For comparison, the catalytic performance of w-CsPbBr_3_ NCs has also been carried out. The results revealed that its reaction yield of CH_4_ (11%), and CO (19%) at − 1.1 V was much lower than that of w-CsPbBr_3_ NCs (Fig. S11a). Moreover, a 34% deduction of the current density for h-CsPbBr_3_ has been observed after 20 h (Fig. S11b). While, the current density for w-CsPbBr_3_ can preserve about 97% of its initial value after 20 h (Fig. 5c), which suggested much higher catalytic stability of w-CsPbBr_3_ than that of h-CsPbBr_3_.

**Fig. 6:**
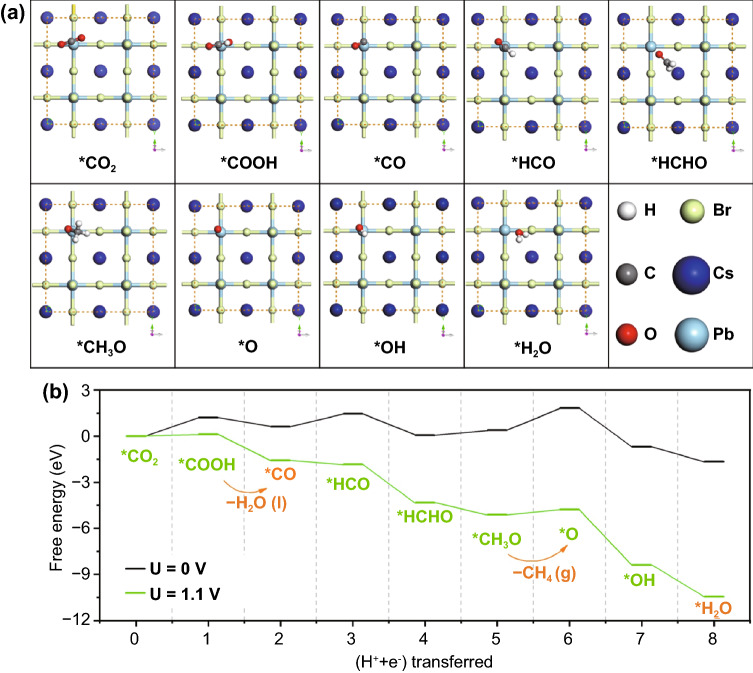
Formation energy of intermediates. **a** Binding site configurations for the CsPbBr_3_ catalysts (**b**) Energy profiles of all intermediates toward CO and CH_4_. Intermediates for all the binding sites on the Pb sites are calculated and then averaged. The energy profiles are corrected to − 0.0 V and − 1.1 V vs. RHE according to the computational hydrogen electrode

Further theory calculations were carried out to investigate the active site and the reaction mechanism of this reaction. Generally, the appropriate bonding energy between CO_2_ molecular and active sites is necessary for the CO_2_ reduction. Our theoretical results in Fig. S12 suggest that the formation of Cs–O bond is preferred to Cs–C bond. In this regard, the Cs cannot be the active site in the CsPbBr_3_ NCs-based electrochemical CO_2_ reduction reaction. However, once we choose the Pb atom as the active site, the formation of Pb–C chemical bond is favourable, as a result, the CO_2_ can be reduced to CO and CH_4_ (Fig. 6). These results indicate that the catalytic center should be the Pb atoms in the perovskite structure., the C–C coupling pathway between two C1 species into one C2 product was substantially inhibited, leading to a dramatic enhancement of the CH_4_ formation (*i.e.*, the deepest C1 product).

## Conclusions

In summary, water-dispersible CsPbBr_3_ NCs with ultra-stability were successfully prepared through a Pb-poor hot-injection method combined with a well-designed purification process. The as-prepared water-dispersible CsPbBr_3_ NCs exhibited a higher PLQY (91%) and lower defect density than the traditional hexane-dispersibility CsPbBr_3_ NCs. Interestingly, the water-dispersible CsPbBr_3_ NCs showed ultra-stability of 200 days with only a ~ 20% decline in the initial PL intensity. CsBr salt can passivate the surface defects induced by a loss of OLA^+^ and Br^−^ ions, and maintain the stability of the CsPbBr_3_ NCs structure in water. Finally, for the first time, CsPbBr_3_ NCs showed high electrocatalytic activity and stability (> 350 h) for the CO_2_ reduction reaction, providing high generation values of CH_4_ (32%) and CO (40%). This work not only provides an effective method for the synthesis of water-dispersible perovskite NCs, but also shows excellent potential for their application in electrocatalysis.

## Supplementary Information

Below is the link to the electronic supplementary material.

Supplementary file1 (PDF 1743 kb)
