# Supplementary material for: Water-Dispersible CsPbBr3 Perovskite Nanocrystals with Ultra-Stability and its Application in Electrochemical CO2 Reduction
Source: Nanomicro Lett. 2021 Aug 12;13:172. doi: 10.1007/s40820-021-00690-8 (PMC8360258; doi:10.1007/s40820-021-00690-8)
Supplement: Supplementary file 1 — Supplementary file1 (PDF 1743 kb) [file 40820_2021_690_MOESM1_ESM.pdf]

Supporting Information for

Water-Dispersible $CsPbBr_3$ Perovskite Nanocrystals with Ultra-Stability and Its Application in Electrochemical CO_2 Reduction

Keqiang Chen[1, 2], Kun Qi[1], Tong Zhou[3], Tingqiang Yang[1], Yupeng Zhang[1, *], Zhinan Guo[1], Chang-Keun Lim[2, 4], Jiayong Zhang[5], Igor Žutić[3], Han Zhang[1, *], Paras N. Prasad[2, *]

[1]Key Laboratory of Optoelectronic Devices and Systems of Ministry of Education and Guangdong Province, Institute of Microscale Optoelectronics, Shenzhen University, Shenzhen 518060, P. R. China

[2]Institute for Lasers, Photonics, and Biophotonics, Department of Chemistry, University at Buffalo, State University of New York, Buffalo, NY 14260, USA

[3]Department of Physics, University at Buffalo, State University of New York, Buffalo, NY 14260, USA

[4]Department of Chemical and Materials Engineering, School of Engineering, Nazarbayev University, Nur-Sultan City 010000, Kazakhstan

[5]Jiangsu Key Laboratory of Micro and Nano Heat Fluid Flow Technology and Energy Application, School of Physical Science and Technology, Suzhou University of Science and Technology, Suzhou, 215009, China

*Corresponding authors. E-mail: ypzhang@szu.edu.cn (Y.Z.), hzhang@szu.edu.cn (H.Z.), pnprasad@buffalo.edu (P.N.P.)

Supplemrntary Figures and Tables

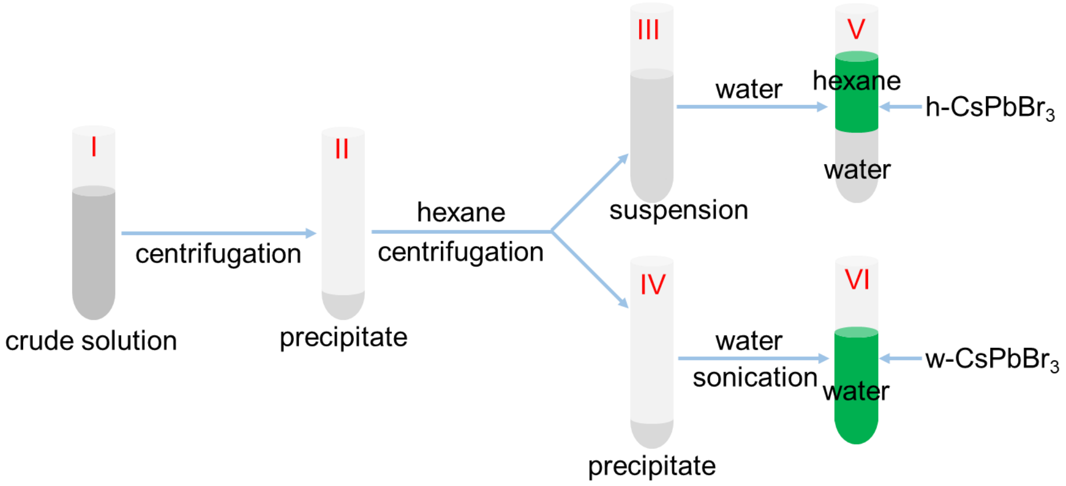

Fig. S1 Schematic of the purification process for the preparation of water-dispersible $CsPbBr_3$ nanocrystals. The as-synthesized curde solution (marked as I) was directly centrifuged at 9000 rpm for 10 min. The suspension was discarded, and the precipitate

(marked as II) was re-dispersed by 10 mL hexane. Subsequently, another centrifugation process was carried out at 9000 rpm for 10 min, the suspension and the precipitate (nonluminescent) were marked as III and IV, respectively. Then, 2 mL of water was mixed separately with III and IV, which were marked as V and VI. After sonication, strong PL emission can be found from the oil phase (dispersed in hexane, makred as h-$CsPbBr_3$) and water phase (dispersed in water, makred as w-$CsPbBr_3$), respectively.

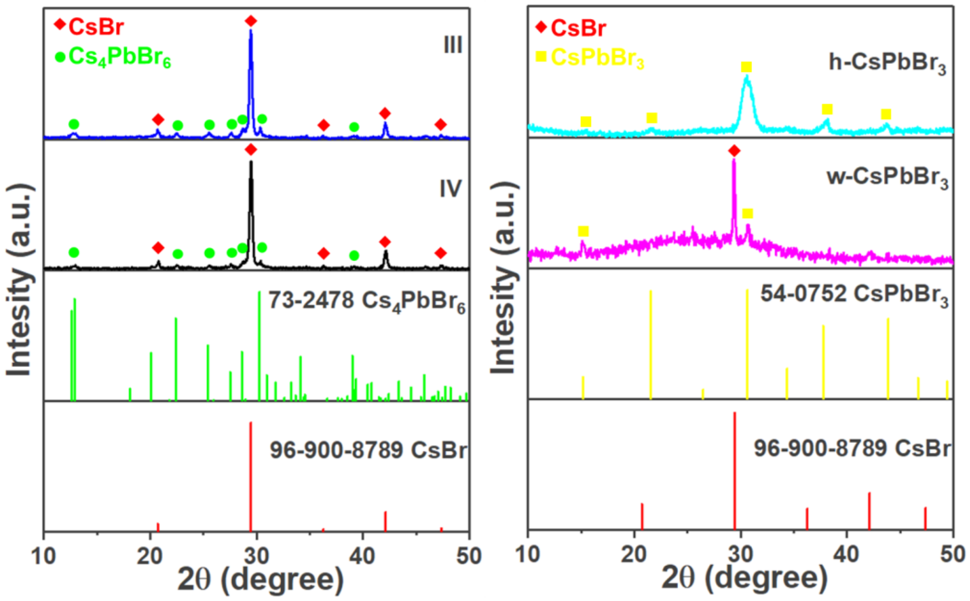

Fig. S2 XRD patterns of **III**, **IV**, **V** (h-$CsPbBr_3$), and **VI** (w-$CsPbBr_3$), which can be indexed as a mixture of CsBr/Cs_4PbBr_6 (**III** and **IV**), $CsPbBr_3$ (h-$CsPbBr_3$) or a mixture of CsBr/$CsPbBr_3$ (w-$CsPbBr_3$), respectively

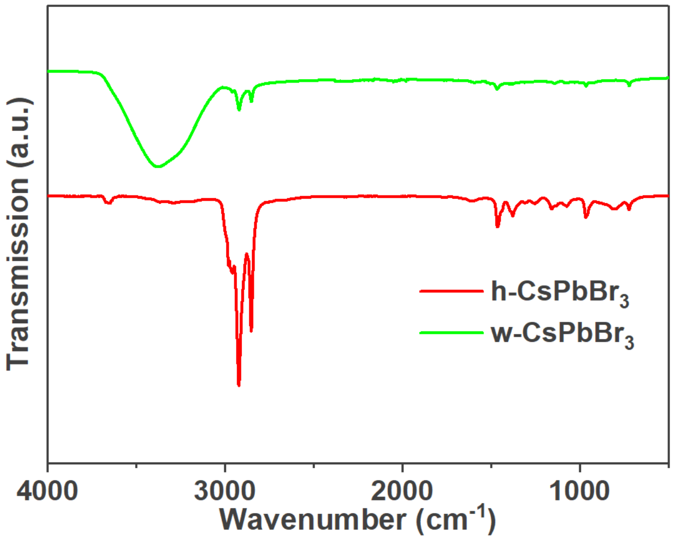

Fig. S3 FTIR spectroscopy of h-$CsPbBr_3$ (red) and w-$CsPbBr_3$ (green)

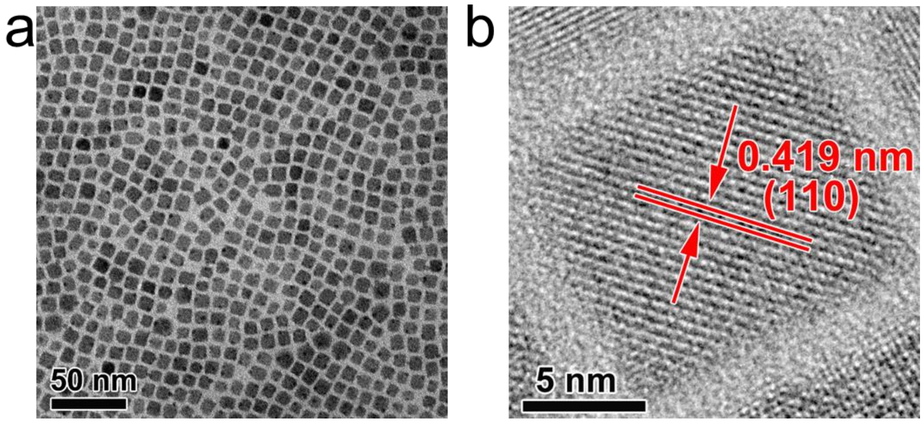

Fig. S4 TEM (**a**) and HRTEM (**b**) images of h-$CsPbBr_3$

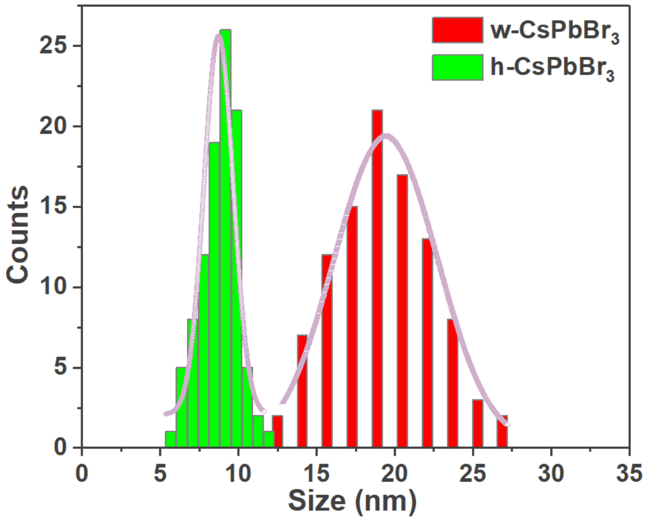

Fig. S5 Size distribution histograms of h-$CsPbBr_3$ and w-$CsPbBr_3$ NCs. The data were collected from 100 NCs for each sample

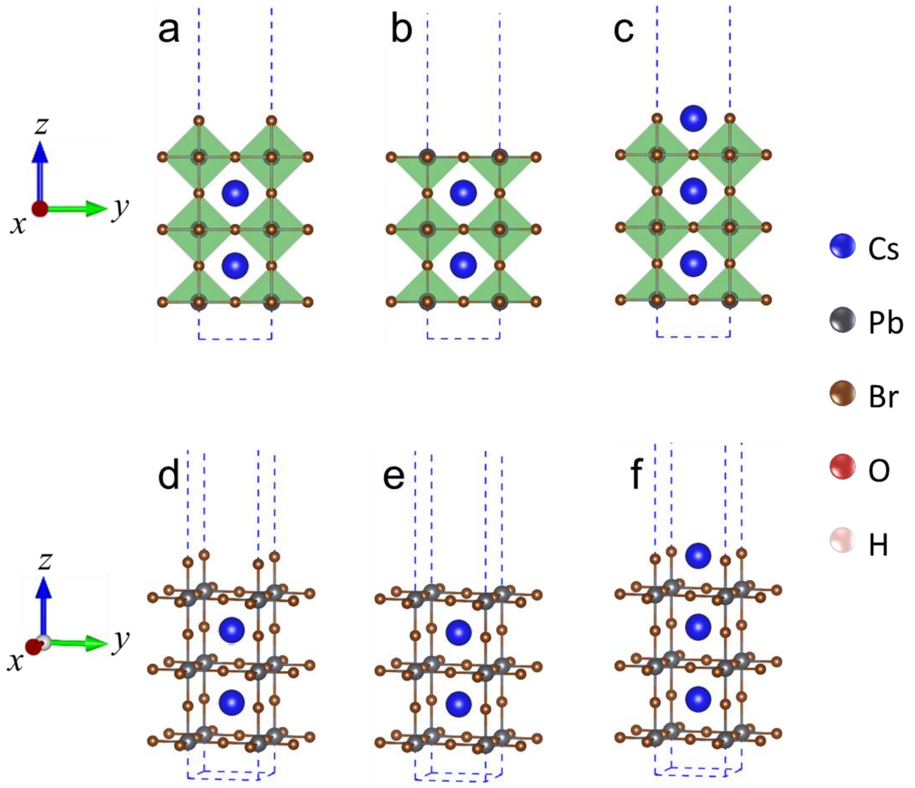

Fig. S6 Unrelaxed (original) polyhedral and ball-and-stick models of $CsPbBr_3$ with PbX_2 terminated surface: (**a**) (**d**) initial structure; (**b**) (**e**) Br-vacancy structure; (**c**) (**f**) CsBr passivated structure. Blue, gray, brown, red, and pink spheres represent Cs, Pb, Br, O, and H atoms, respectively

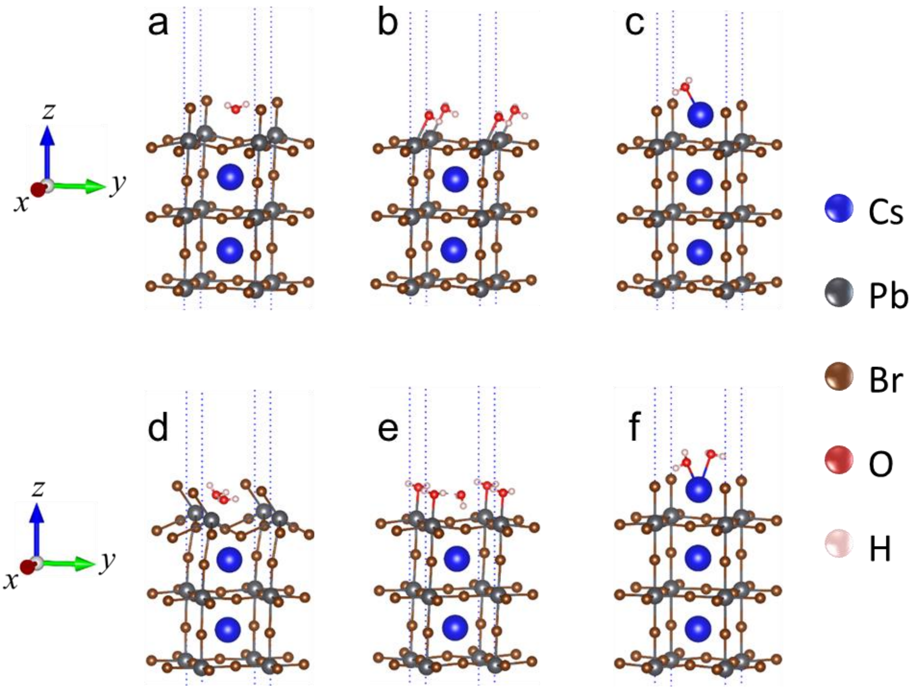

Fig. S7 Relaxed ball-and-stick models of $CsPbBr_3$ with various structures after adsorbing one or two water molecules: (**a**) initial structure with one water molecule; (**b**) Br-vacancy structure with one water molecule; (**c**) CsBr passivated structure with

one water molecule; (**d**) initial structure with two water molecules; (**e**) Br-vacancy structure with two water molecules; (**f**) CsBr passivated structure with two water molecules. Blue, gray, brown, red, and pink spheres represent Cs, Pb, Br, O, and H atoms, respectively

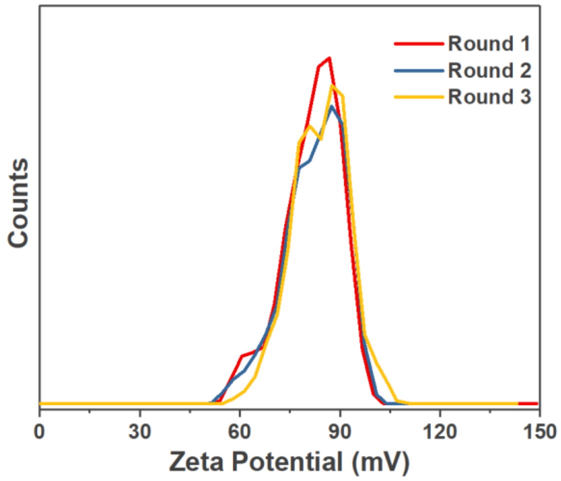

Fig. S8 Zeta potential of $CsPbBr_3$ NCs solution

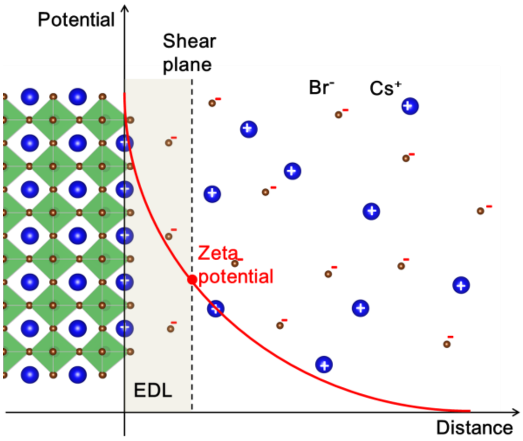

Fig. S9 Schematic graph of the EDL around the surface of $CsPbBr_3$ NCs

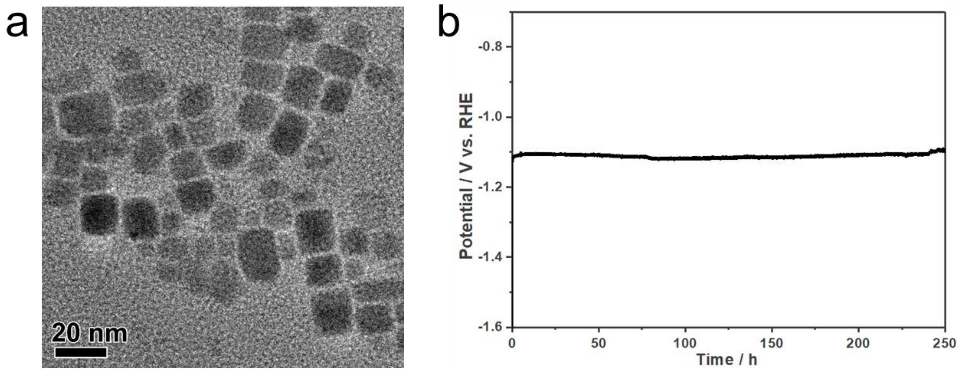

Fig. S10 (**a**) TEM image of the $CsPbBr_3$ NCs after the CO_2 reduction reaction; (**b**) CP curve of the $CsPbBr_3$ NCs with a constant current density of 25mA cm^{-2}

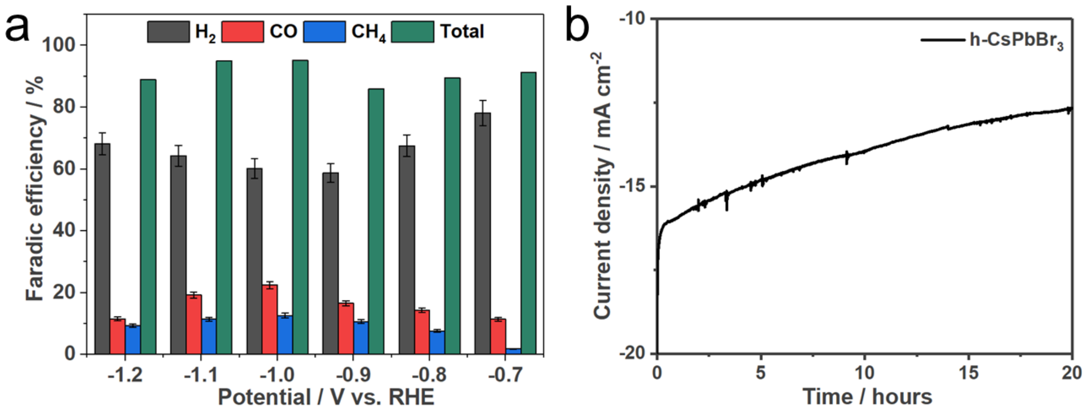

Fig. S11 (**a**) Faradic efficiencies for the h-CsPbBr$_3$ NCs CO_2 RR; (**b**) chronopotentiometry curve (long-term stability) of h-CsPbBr$_3$ NCs in 0.1 M KHCO$_3$ for 20 hours

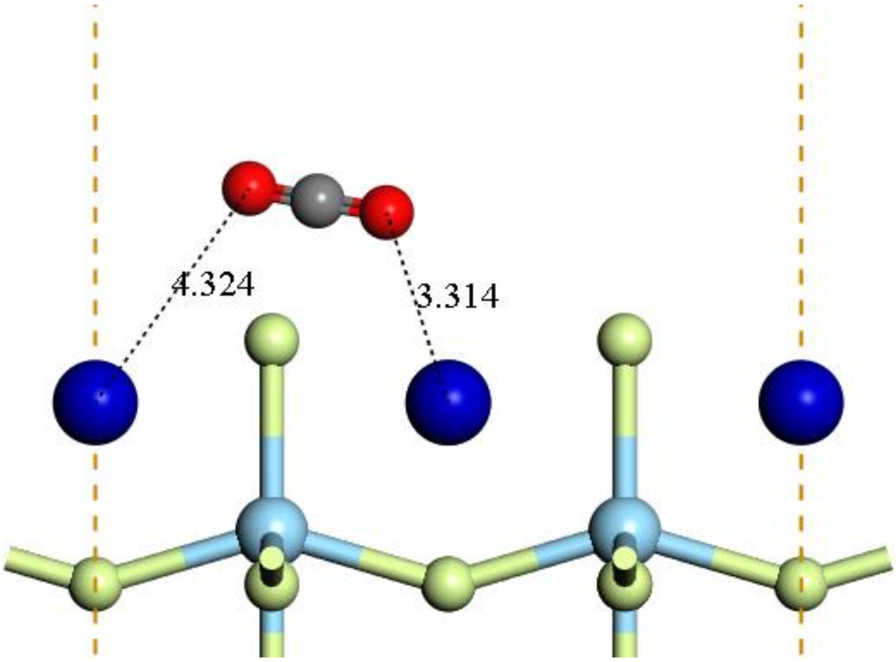

Fig. S12 Binding configurations between Cs atoms and CO_2

Table S1 Statistics of the deviations of the Pb−Br bond lengths and the Pb−Br−Pb bond angles of $CsPbBr_3$ models under the adsorption of water molecules. The crystal structure and bond angles are difined as following figure:

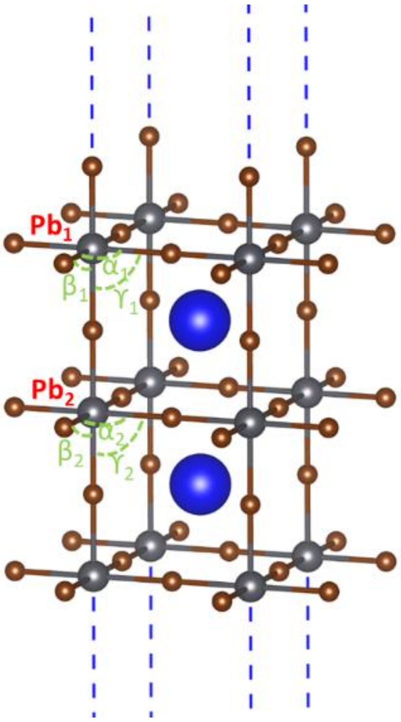

	No. of water	Position	Pb-Br bond length (Å)						D* (Å)
			left	right	up	down	front	behind	
Initial structure	/	1st layer	2.937	2.937	2.937	2.937	2.937	2.937	0
		2nd layer	2.937	2.937	2.937	2.937	2.937	2.937	0
$PbBr_2$ terminated (Figure 4a and 4g)	One water	1st layer	3.158	2.851	2.683	3.258	2.983	3.002	0.196
		2nd layer	2.85	3.035	2.823	3.094	2.91	2.977	0.097
	Two water	1st layer	3.677	2.848	2.885	3.786	2.903	3.379	0.496
		2nd layer	2.949	2.94	2.788	3.139	2.952	2.94	0.103
Br-vacancy (Figure 4b and 4h)	One water	1st layer	3.058	2.85	NA	2.942	3.05	2.839	0.094
		2nd layer	2.946	2.952	3.025	3.042	2.939	2.94	0.056
	Two water	1st layer	3.064	2.768	NA	3.068	2.921	2.983	0.113
		2nd layer	2.96	2.968	3.073	3.075	2.957	2.968	0.082
CsBr passivated (Figure 4c and 4i)	One water	1st layer	2.941	2.948	2.984	2.982	2.943	2.946	0.027
		2nd layer	2.937	2.938	2.948	2.991	2.937	2.937	0.023
	Two water	1st layer	2.934	2.952	2.998	2.983	2.944	2.941	0.032
		2nd layer	2.939	2.935	2.947	2.997	2.937	2.937	0.025

* Standard deviation from the original Pb-Br bond length.

	No. of water	Pb-Br-Pb bond length (○)							
		α_1	β_1	Υ_1	D* (Å)	α_2	β_2	Υ_2	D* (Å)
Initial structure	/	90.000	90.000	90.000	0.000	90.000	90.000	90.000	0.000
$PbBr_2$ terminated (Figure 4a and 4g)	One water	91.777	79.06	80.109	8.577	88.687	86.646	87.186	2.639
	Two water	99.136	76.106	71.53	14.348	89.812	85.345	87.29	3.112
Br-vacancy (Figure 4b and 4h)	One water	89.927	84.625	85.679	3.982	90.026	89.503	91.929	1.15
	Two water	93.73	83.708	84.579	5.256	88.012	88.284	94.861	3.19
CsBr passivated (Figure 4c and 4i)	One water	89.797	86.57	86.928	2.661	89.866	90.378	90.151	0.248
	Two water	89.402	86.503	86.912	2.715	90.125	90.498	90.583	0.448

* Standard deviation from the original Pb-Br-Pb bond angle.
